# Supplementary material for: Examining the challenges in sustaining user engagement with a mobile app to enhance multidrug-resistant tuberculosis (MDR-TB) care in Vietnam and its implications for implementing person-centred mHealth interventions
Source: PLOS Glob Public Health. 2025 Apr 16;5(4):e0004454. doi: 10.1371/journal.pgph.0004454 (PMC12002538; doi:10.1371/journal.pgph.0004454)
Supplement: Attachment — Submitted filename: VSMART Qual_Response to Reviewers.docx [file pgph.0004454.s003.docx]

**Response to Reviewer and Editor Comments – Making it fit for (everyone’s) purpose: Examining the challenges of capitalising on mHealth’s potential to deliver locally-aligned person-centred care for drug resistant tuberculosis in Vietnam**

Dear Editors,

We sincerely appreciate the time and effort you and the reviewers have dedicated to evaluating our manuscript titled ‘*Making it fit for (everyone’s) purpose: Examining the challenges of capitalising on mHealth’s potential to deliver locally-aligned person-centred care for drug resistant tuberculosis in Vietnam’* (subjected to change as per reviewer’s comment) (ID: PGPH-D-24-02013). We are grateful for the insightful comments and constructive feedback, which have helped us improve the clarity and rigour of our work.

In response to the comments provided, we have carefully revised the manuscript and addressed each point raised by the editor and reviewers. Below, we have provided a detailed, point-by-point response, outlining the changes made and the rationale behind them. For easy tracking, we have included the page and line numbers (in the unmarked version) where changes were made to address each comment.

We hope that our revisions have satisfactorily addressed the concerns raised, and we appreciate your consideration of our revised manuscript. Please do not hesitate to reach out if any further clarification is needed.

Thank you once again for your thoughtful review. We look forward to your feedback.

| **No** | **Comment/Request** | **Response** |
| --- | --- | --- |
| **Journal Requirements** | | |
| **1** | Please include a complete copy of PLOS’ questionnaire on inclusivity in global research in your revised manuscript. Our policy for research in this area aims to improve transparency in the reporting of research performed outside of researchers’ own country or community. The policy applies to researchers who have travelled to a different country to conduct research, research with Indigenous populations or their lands, and research on cultural artefacts. The questionnaire can also be requested at the journal’s discretion for any other submissions, even if these conditions are not met.  Please find more information on the policy and a link to download a blank copy of the questionnaire here: [https://journals.plos.org/globalpublichealth/s/best-practices-in-research-reporting](https://url.au.m.mimecastprotect.com/s/RuI3CQnMBZfN4yM2RTPh5IGHAC6?domain=journals.plos.org). Please upload a completed version of your questionnaire as Supporting Information when you resubmit your manuscript. | Thank you. The questionnaire on inclusivity in global research has now been completed and submitted to the portal. |
| **2** | Please send a completed 'Competing Interests' statement, including any COIs declared by your co-authors. If you have no competing interests to declare, please state "The authors have declared that no competing interests exist". Otherwise please declare all competing interests beginning with the statement "I have read the journal's policy and the authors of this manuscript have the following competing interests:" | We have now provided an additional statement to the 'Competing Interest' section on the submission portal. |
| **3** | 3. In the online submission form, you indicated that "The data used in this study cannot be shared publicly as it violates the agreement to which the participants consented. Data will only be made available on request.".  All PLOS journals now require all data underlying the findings described in their manuscript to be freely available to other researchers, either  1. In a public repository,  2. Within the manuscript itself, or  3. Uploaded as supplementary information.  This policy applies to all data except where public deposition would breach compliance with the protocol approved by your research ethics board. If your data cannot be made publicly available for ethical or legal reasons (e.g., public availability would compromise patient privacy), please explain your reasons by return email and your exemption request will be escalated to the editor for approval. Your exemption request will be handled independently and will not hold up the peer review process, but will need to be resolved should your manuscript be accepted for publication. One of the Editorial team will then be in touch if there are any issues. | Thank you for your notice regarding the data availability policy. The data used in our study is highly identifiable and sensitive; therefore, making it publicly available would violate the terms of the informed consent provided by participants. Our study protocol (number 2019/676), approved by The University of Sydney Human Research Ethics Committee (HREC) and Vietnam National Lung Hospital Institutional Review Board (number 13/19/CT-HDDD), explicitly states that data will only be shared upon reasonable request in a manner that ensures participant confidentiality, except where required by law.  We kindly request an exemption from the data-sharing requirement on ethical grounds. Please let us know if any further details are needed to process this request. |
| **4** | 4. Please provide an Author Summary. This should appear in your manuscript between the Abstract (if applicable) and the Introduction, and should be 150–200 words long. The aim should be to make your findings accessible to a wide audience that includes both scientists and non-scientists. Sample summaries can be found on our website under Submission Guidelines:  [https://journals.plos.org/globalpublichealth/s/submission-guidelines#loc-parts-of-a-submission](https://url.au.m.mimecastprotect.com/s/Eei1CROND2ugPzjMOfPigI1Dn_g?domain=journals.plos.org) | We have now included the author summary in the manuscript.  The additional text can be found on pages 3 and 4, lines 5-71: ‘’ There has been considerable interest in the potential of Mobile health (mHealth) apps to improve the delivery of care for patients with health conditions, such as multi-drug-resistant Tuberculosis (MDR-TB), which require intensive support. In Vietnam, a smartphone app designed to support improved the timely reporting and responsive management of the side effects, known as adverse events (AE), of MDR-TB treatment has been tested. We conducted 37 interviews with patients and healthcare workers to understand why their engagement with the app declined over time. Initially, patients were motivated to use the app, seeing it as a promise of 'good care' from trusted healthcare workers, however their expectations of the functionality of the app were not met. HCWs similarly found it had limited utility, and it increased their workload. Over time they reverted to prior forms of communication, like phone calls and in-person visits, but with increased reporting of AE. Our study highlights that we can misattribute initial enthusiasm to use an app as indicating its acceptability and usefulness. We propose a framework that encourages researchers and implementors to explain why people use an app, attending to the social as well as functional reasons. This is vital to help us design and evaluate mHealth technologies that meet local needs and can be successfully integrated to work within health systems.’’ |
| **5** | 5. Figures 1 and 2: Please confirm whether you drew the images / clip-art within the figure panels by hand. If you did not draw the images, please provide (a) a link to the source of the images or icons and their license / terms of use; or (b) written permission from the copyright holder to publish the images or icons under our CC-BY 4.0 license. Alternatively, you may replace the images with open-source alternatives. See these open source resources you may use to replace images / clip-art:  - [https://commons.wikimedia.org](https://url.au.m.mimecastprotect.com/s/UdsyCVARKgCPjRNWZCJslIEfYBx?domain=commons.wikimedia.org)  - [https://openclipart.org/](https://url.au.m.mimecastprotect.com/s/eAwnCWLVXkUywWRM4Cmt3IoSJXE?domain=openclipart.org) | We have now removed Figure 1 to ensure that there is no risk of it violating intellectual property as suggested in the following comment. We have also updated Figure 2 as the original one contained a mobile app interface screenshot.  Figure 2 is our original illustration generated with Microsoft PowerPoint. |
| **6** | 6. Figures 1 and 2 contain screenshots. We are not permitted to publish these under our CC-BY 4.0 license; websites are usually intellectual property and are copyrighted. This includes peripheral graphics of the web browser such as the [X] buttons. We ask that you please remove or replace it. | Thank you for the information. We have now removed Figure 1 entirely and the screenshot in Figure 2. Figure 2 is now Figure 1 in the revised manuscript. |
| **Reviewer 1** | | |
| 7 | I want to commend the authors for the ambitious project that they have undertaken with this manuscript. It is well-written and will provide valuable insights to the field. I hope that the following comments can be used to improve the manuscript. | Thank you for valuing the contribution of our paper. |
| 8 | I received a PDF without numbered lines, so I have listed page numbers to help locate the specific areas of the text. In the next version, please follow the journal’s submission guidelines and include continuous line numbers. | Apologies, the updated manuscript includes continuous line numbers. |
| 9 | The title does not seem to perfectly align with the ambitions of the manuscript. This decision is entirely up to the authors, and I will not reject the paper for this reason, but a modified title would probably be clearer to readers. | We agree that the original title did not sufficiently capture the extended scope of the paper.  We have updated the title to reflect the adjustments made to the paper on page 1 lines 1-4: ‘Examining the challenges in sustaining user engagement with a mobile app to enhance multidrug-resistant tuberculosis (MDR-TB) care in Vietnam and its implications for implementing person-centred mHealth interventions.'' |
| 10 | The abstract should be revisited by the authors. The study design was insufficiently explained in the abstract. It did not make clear how this study fit within a larger clinical trial. It was unclear if this was an exploratory or explanatory qualitative study (or a combination) nested within a trial. Page 7 and the conclusion describe a “case study” which was also not mentioned. Neither the qualitative analysis method nor the development of a conceptual framework were included in the abstract. | We have revised the abstract as per the reviewer’s suggestions. We also agree that the original abstract was insufficient in terms of method and caused confusion.  In response to the reviewer's question about whether the study is explanatory exploratory or both, our study aimed to explain (we have used the word 'understand') the observed reduction in the use of the app, but inherent in in-depth qualitative research is an exploration of participant experiences to increase understanding.  The updated abstract now includes sentences about the study design and analysis method in the abstract to address the points you made on page 2 lines 32-37: ‘’This **qualitative** study examines user experiences to **explain** reported reduction in engagement with (i.e. use of) a smartphone app which is **being trialed** to improve early detection and management of adverse events (AEs) among MDR-TB patients in Vietnam (VSMART trial). We conducted 37 in-depth interviews with patients and healthcare workers (HCWs) and **thematically analyzed** the data.’’  We have included another sentence second to the last sentence in the abstract to indicate our conceptual framework on page 3, lines 47-51: ‘’To support attention to this process, we propose an **explanatory** framework that captures the social-functional dynamics of mHealth interventions which can guide much needed qualitative evaluation to support the design of mHealth technologies to align with stakeholders’ needs and suited for integration into local healthcare systems.'' |
| 11 | Citation 5 should cite the most recent GTB report. | The reference we previously used was indeed outdated. Thank you.  We have now updated the citation which is now citation number 6 on page 5, line 88; and the corresponding reference on page 39, line 715 to ‘’Organization WH. Global Tuberculosis Report 2024. World Health Organization; 2024. 69 p''. |
| 12 | In the description of the V-SMART trial, it is unclear how people were selected to receive access to a smartphone and data packages. How was socioeconomic status assessed to determine equitable access to technology? What proportion of individuals received data and smartphones among those who participated in the quali sample? The amount of data provided monthly was unclear along with the expectations/terms for receiving & returning a loaned phone. These financial motivators could have a large impact on participants and should not be ignored. It could be argued that among the ‘performative use’ group, their performative participation in the project was in exchange for the financial return of participation, in an even more direct way than engaging with a symbol of good care. Also, among the unproductive use group, were these people provided with smartphones from the project and then insufficiently trained on how to use them? Did they pass this new asset to their kids and grandkids to provide an additional resource to the household, but then they stopped engaging with the app and continued to use the phone? These financial motivations for participation should be explored in greater detail within the manuscript. | Thank you for prompting us to clarify this issue. After consulting again with the trial team, we have updated information regarding data packages and smartphones in the Introduction.  We did consider the financial aspects of app use due to the way VSMART was designed and implemented. However, no patient participants mentioned the incentive as the motivation for participation despite being asked.  In addition, the data incentive for trial participants was relatively small, as was the number of participants who received a loaned smartphone compared to the overall trial sample. While we acknowledge that financial factors may have played a role, identifying or isolating their impact from the already complex web of influences was particularly challenging within our already limited qualitative sample.  We have added details on the data incentive package in the VSMART trial and the number of participants in the control arm who have received loaned phones to date on page 10, lines 183-188: ‘’Given that participation relied on access to a smartphone, participants (both patients and HCWs) receive a small monthly data allowance (70 000 VDN/approximately 4.40 AUD) which was paid as a reimbursement. A smartphone was loaned to sixteen intervention arm participants (3.56%) who did not have their own or whose smart phone was not compatible with the app, one of whom participated in the qualitative study.''  We have also included sentences to acknowledge the absence of the financial factor identified and suggest how it could be addressed in future research in the limitation section on page 37 lines 679-681: ‘The financial impact on app use is under-explored. Future research should examine the role of financial incentives on app engagement.‘’ |
| 13 | Additionally, it is unclear in the introduction how or why the app would provide the personal phone numbers of the HCWs to the patients. “I only give my personal number to patients who tend to have more problems with medication. But now most of the patients [with the app] can access my number, they now text me too, I have to answer… it is good to help patients contact the senior doctor directly, but it becomes too much for me to handle.’’ Was it a design flaw that provided phone numbers to participants instead of centralizing communication within the app? Did the HCWs consent to having their number distributed prior to the study? | We thank the reviewer for prompting us to provide background to this issue.  The issue raised by the reviewer stems from a design flaw in the app. VSMART trial participants were required to sign up using their phone numbers. HCWs were asked to provide their phone number to the trial given that it was their phone number that would connect their patients’ app to theirs. As such, they consented to the use of their phone number. Study staff entered the HCW’s phone number into the patient’s app to set it up. HCWs phone number meant to be concealed and inaccessible within the app. However, once a patient was paired with their healthcare worker, they could see the healthcare worker’s phone number in the information section.  Our qualitative study identified this design flaw and flagged it to the trial team for modification.  We have now added extra details to the VSMART trial description in the introduction to contextualize the issue in the result. First, additional text on the function of the phone number on page 9 lines 167-168: ‘’Trial participants - patients and HCWs - are requested to register an app account using their phone number (as unique identifier).’’  We have also added a sentence that makes clear the design issue is identified by the qualitative evaluation on page 30 lines 539-540: ‘It was in part enabled by a flaw we identified in the app’s design that HCWs’ phone numbers were visible to patients.’’ and on page 34 lines 631-633: ‘’Accounting for social relationship dynamics among different user groups helps identify implementation risks which are often overlooked in evaluations focusing on a single type of user.’’ |
| 14 | The design section needs some clarification. What is the precise study design? Is it a qualitative study or a qualitative case study? If it is the former, then I would recommend adhering to the Consolidated criteria for reporting qualitative research- the COREQ checklist- and mentioning your methodological orientation. If it is the latter, then I would recommend grounding it in other research on case study designs. For example: Priya, A. (2021). Case Study Methodology of Qualitative Research: Key Attributes and Navigating the Conundrums in Its Application. Sociological Bulletin, 70(1), 94-110. | The study design is strictly qualitative. Our use of the term "case" was intended to highlight our contribution to the broader literature on mHealth interventions. We acknowledge that our previous description of the methods lacked sufficient detail in accordance with the Consolidated Criteria for Reporting Qualitative Research (COREQ).  We have made several changes and additions to address these issues:   (1) We have removed ''case study'' and changed to 'qualitative study'' in relevant parts including the abstract, method and conclusion in the manuscript. (2) Additional details have been provided in the Methods and Results sections to better align with the COREQ checklist. Each domain is addressed, with sub-domains italicized and placed in square brackets as signposts before the relevant text:  - Domain 1 - Research team and reflexivity:   Additional text on page 12 lines 225-229: ‘’ *[personal characteristic of interviewer]* Interested patients were contacted by phone by a Vietnamese qualitative researcher (DT, male) to discuss the qualitative study further. The primary qualitative researchers working on this study (SB, DD and DT) were not members of VSMART trial team and did not have prior relationships with potential participants.’’   - Domain 2 - Study design:   Additional text on pages 11-12, lines 210-220:‘’*[Participant selection - Sampling]* Our selection was informed by the key sampling variables of sex, age, and urban/peri-urban/rural residence, within this we also sought to include trial participants whose app usage reflected the two dominant patterns identified within the quantitative trial data: 1) those who had stopped using the app after a period of consistent engagement including those who used the app once or only a few times upon enrolment, and 2) those who used the app sporadically across their treatment. Sampling concluded when we had reached thematic saturation and when we were satisfied that the distribution of participant characteristics across the conceptual framework reflected established patterns of risk factors for TB (6) and expected associations between patient characteristics and use of novel technologies.’’  Additional text on pages 14, lines 261-262: ‘’[Analysis and Findings] Thematic analysis was conducted, an approach well suited to understanding nuanced issues surrounding use and implementation of mHealth (33).’’  Additional text on pages 14, lines 263-266: ‘*[Data collection - Data saturation]* The qualitative research team met after every 2-3 interviews to discuss the interview summaries in-depth, which informed both a refined interview guide and further sampling decision (38).’’  Additional text on page 15, lines 270-278: ‘’*[Number of data coders, Software, Derivation of themes]* DD and DT collaboratively developed preliminary codes from interview summaries. Once the analysis team reached a consensus, the codes were applied to the full transcripts using Microsoft Word. They were progressively refined and organized, within Microsoft Excel, into key themes (39) linkages between patterns of app use, participant characteristics, and key themes increasingly honed. This iterative process of interpretation and conceptualization produced a conceptual framework inductively (40) to explain the observed reduction of app use among patients, which we present within an overall explanatory narrative (41).’’   - Domain 3 - Analysis and findings:   Additional text on page 14, lines 255-258 ‘’*[Data analysis -Participant checking]* In later interviews we discussed our emerging interpretations, as a form of validation. These were also presented to trial staff to relay back the implementation challenges they encountered by patients and HCWs.’’  Additional text in the result section, on pages 16-17, lines 289-295 1: **‘’***[Clarity of minor and major themes]* Although our results are presented sequentially, Fig 1 illustrates inter-related nature of the results. We first describe the pre-existing standard of care for managing AEs to characterize the ‘relational care space’ into which the mHealth intervention was introduced. We then examine the experiences of using it from the perspective of HCWs, and patients to identify the key moderating factors shaping the app’s uptake and use. We unpack the consequences, including those that were unintended, of the app in the final section.’’, and Figure 1 on page 17. |
| 15 | The aim of the paper does not appear to be consistent throughout. You wrote, “The qualitative evaluation aimed to adapt the app to better align with the needs of the trial’s end users.” There is some uncertainty about whether this paper primarily focuses on evaluating the Bac Sy Minh app to improve its use for MDR-TB or to use this local case study to develop a broader iterative approach to evaluate mHealth interventions. The development of a conceptual framework and the presentation of the results and discussion seem to aspire to the broader ambition, but this is not included in the stated aim. Please clarify. | We appreciate the reviewer’s insightful comment. This paper does not report on changes made to the app. Instead, this paper focuses on presenting findings that illustrate an iterative approach to evaluating mHealth interventions over time (results). Additionally, we provide practical recommendations on key priorities for developing mHealth apps for MDR-TB (discussion).  Although an objective of this study was to gather user feedback, which was compiled into an internal report for the trial team to inform app modifications (related to issues raised in Comment 13). However, this aspect has now been removed and de-emphasized in the paper to maintain focus on the study’s primary aims. Thank you for pointing out the confusion.  We have removed the statement: “The qualitative evaluation aimed to adapt the app to better align with the needs of the trial’s end users.”  We have clarified the aim of our paper in the Introduction on page 8, lines 140-143: ‘’ This qualitative study sought to explain why although there was initially high uptake, there was an unexpected decline in app engagement (use), with the proportion of patient users checking in at least 5 days/week dropping from 58.6% in the first month (October 2020) to 8.3% by the eleventh month (forthcoming publication).’’  We have also corrected relevant text at the beginning of the Discussion section on page 31, lines 561 and 563: ‘’We qualitatively investigated the reduction in user engagement with an mHealth app being trialed to enhance the management of AEs within routine MDR-TB care in Vietnam.’’ |
| 16 | Then you wrote, “We used individual in-depth interviews to understand…the app’s acceptability…” This manuscript does not sufficiently engage with the topic of acceptability. The only point the paper seems to explicitly make about acceptability is that the social is a mediator of acceptability. I could not discern a working definition of acceptability in the manuscript. The word acceptability does not appear in the results. Would the Bac Sy Minh app even be considered acceptable? A commonly cited theoretical framework of acceptability (Sekhon, M., Cartwright, M. & Francis, J.J. Acceptability of healthcare interventions: an overview of reviews and development of a theoretical framework. BMC Health Serv Res 17, 88 (2017).) proposes multiple constructs forming an understanding of acceptability. You do not need to accept this suggestion, but its concept of ‘intervention coherence’ may help explain how misunderstandings of the “remit of the app” lowered its acceptability and how the added burden placed on the HCWs could also diminish acceptability. | We appreciate that the reviewer pointed out the confusion and provided valuable suggestions for consideration. This comment is in part related to Comment 15 regarding the consistency of the aim of the paper. Indeed, it was not an aim of our paper to assess and report on acceptability. To clarify, we have modified this focus on acceptability in the introduction and abstract to make explicit that our focus is concentrated on engagement/use. Rather consideration of acceptability as it emerged in our analysis informed our understanding of the dislocation between how the app was intended to be used and how it was used in practice.  In addition to clarifying the focus of the paper (Comment 15), we have also edited and added to the introduction to delineate acceptability and engagement in relation to mHealth effectiveness to preemptively signpost our research focus on engagement (use).  To ensure that clear connections are being made back to the broader literature, we have also reflected on how our findings focusing on engagement with mHealth technologies/intervention bear relevance to the understanding of mHealth acceptability when considering effectiveness. We have integrated aspects of the findings that align with elements of Sekhon et al’s framework of acceptability, especially the concept of 'intervention coherence’, into the discussion.  In addition to the edit made in Comment 15, to clarify the focus of the paper, we have provided additional information on current mHealth literature on pages 6-7, lines 113-121: ‘’Acceptability (domains of which include: affective attitudes, intentional use, and pre- and post-use satisfaction) has tended to be considered an initial predictor of mHealth success but empirical studies indicate that it is commonly not sustained (23,28). Digital health researchers call for a more granular exploration of related but distinct concepts of engagement, encompassing behavioral (usage patterns), cognitive (interest) and affective (enjoyment) dimensions to develop a more nuanced understanding of the variation in what underpins initial versus sustained user interaction when considering mHealth intervention effectiveness and widespread adoption (25,29).’’  Discussion on how engagement links to acceptability when considering effectiveness has also been added to pages 33-34, lines 608-618: ‘’Our focus on engagement and use patterns highlighted some valuable dimensions for explaining how acceptability mediates effectiveness (25). Recent research shows how novel digital technologies often do not function as anticipated when implemented into complex healthcare environments and systems (23,51). Ensuring that we adopt a temporal lens enables us to observe critical changes, such as fluctuating uptake and use, over time (25,28). The dislocation that we noted between intended use as designed, and the anticipated functionality among users highlights the criticality of attending to the intervention’s coherence (i.e., how well participants comprehend the intervention and its mechanisms) as defined within Sekhon et al.'s acceptability framework (52) in understanding how initial enthusiasm may not translate into engagement, sustained use and effectiveness for users.’’ |
| 17 | How was a sampling endpoint achieved- data saturation, information power, prior studies? My broader question is, why was purposive sampling for maximum variation stopped with only 2 patients in the functional use and four in the enhanced use groups? | Thank you for highlighting these critical points. Regarding the sampling endpoint and approach, we have addressed this in part in our response to Comment 14. We aimed to recruit the sample to reflect the demographic characteristics of the trial sample and the two dominant patterns of app use.  However, we acknowledge the major concern regarding the small number of participants identified within the functional use category of our conceptual framework. To clarify, we did not intentionally sample fewer participants in certain categories. Rather, our sampling process itself guides the development and emergence of the conceptual framework. In other words, the framework was not predetermined but evolved inductively through sampling and data analysis.  Your comments have given us valuable insight into the reader’s perspective, helping us refine our explanation. Given the descriptive nature of the Methods section, it was challenging to convey this point as clearly as we intended. Instead, we have expanded on this explanation in a more reflective and detailed discussion of the Strength and Limitation section.  We have provided additional text on pages 36-37, lines 659-676: ‘’We also developed a novel conceptual framework that accounts for social drivers of engagement with mHealth technology. Initially our sampling approach focused on recruiting from within two broad groups, higher use participants and low use participants. Within our iterative analysis we identified four explanatory categories of use, which were unevenly distributed across the sample. We oversampled for higher use in an attempt to include more participants displaying functional and enhanced use. However, they remained rare (e.g. only two participants in the functional use category). Even recruiting for specific sampling characteristics that we tentatively considered may be an indicator of these categories, for example high technological literacy, youth, and higher socioeconomic status, did not lead to further identification. With limited study resources, recruitment concluded once we reached thematic saturation, both in terms of consistency within the categories and no further categories of use emerging. The distribution of the sample across the categories, to the best of our knowledge, reflects the broader patterns identified in usage within the trial participants at the time of the qualitative study being conducted. Given that our findings are shaped by the context of this study and the cross-sectional enquiry, we welcome further investigation into its transferability within and beyond TB-focused AE notification apps and the Vietnamese context.’’ |
| 18 | Page 12- Please explain why codes were inductively developed from interview summaries and not transcripts. | We apologize for any confusion caused by the data analysis description in the previous version. We initially developed codes inductively from interview summaries to enable a more efficient analysis while still capturing key themes. These preliminary codes provided a foundation for team discussions and facilitated comparisons with newly recruited participants, rather than delaying analysis until full transcript coding was complete. We then coded the full transcripts using these emerging themes to refine and consolidate them, ensuring a rigorous and well-developed conceptual framework.  We have streamlined and added details to the data analysis sub-section on pages 14-15:   - Lines 266 and 267: ‘These discussions were written up into analytical memos to track early analytical ideas.’ - Lines 270-275: ‘’DD and DT collaboratively developed preliminary codes from interview summaries. Once the analysis team reached a consensus, the codes were applied to the full transcripts using Microsoft Word. They were progressively refined and organized, within Microsoft Excel, into key themes (39) linkages between patterns of app use, participant characteristics, and key themes increasingly honed.’’ |
| 19 | Did the authors complete framework analysis? The type of qualitative analysis is unclear and pages 12 & 13 do not include any citations in the section on analysis. | The confusion likely arose from our misuse of the term “framework matrix,” when we should have originally referred to it as the preliminary code list. We confirm that we only conduct thematic analysis, not framework analysis.  We also agree that there was a lack of citations for qualitative analysis. Thank you for pointing that out.  We have removed the confusing term and revised the analysis process in accordance with Comments 14 and 18. In this section, we have also included multiple citations to clearly indicate the use of thematic analysis throughout the text. |
| 20 | What was done to improve trustworthiness (credibility, transferability, dependability, confirmability) in the qualitative research? | We attempted to enhance the validity of our findings in various ways:   - We sampled from both groups of users (HCWs and patients) which allows triangulation of their accounts. - In later interviews, we relayed back some of our preliminary findings to check if the interpretation of the broader pattern resonated with the experience of the participants. - We periodically had conversations with trial officers who worked closely with participating HCWs and patients. They validated our findings and gave further insight into implementation challenges.   We have added texts in the Method section on page 14, lines 255-258: ‘’In later interviews we discussed our emerging interpretations, as a form of validation. These were also presented to trial staff to relay back the implementation challenges they encountered by patients and HCWs.’’  We have re-emphasized how what has done improved trustworthiness as a strength of this study on page 36, lines 654-658: ‘’This study examines technology-mediated interactions between patients and HCWs which is underexplored in mHealth literature (57,63). Triangulating data from both groups also helped mitigate self-reporting biases.’’ |
| 21 | Page 18- “The focus on adopting a ‘good care’ logic shaped the adaptation of AEs and outpatient care in practice.” The phrase “adaptation of AEs” was a bit unclear. Do you mean AE reporting or how patients perceived their AEs? | Thank you for prompting us to clarify this sentence. We have now revised it, along with the surrounding sentences, to provide a more nuanced and precise description.  The paragraph that previously contained the phrase has now been revised on pages 17-18, lines 311–315: ‘’‘Quan tâm’ encapsulates how patient-participants consistently described their care. This Vietnamese phrase captures the attentive healthcare provided by HCWs. Patients described that they display genuine interest in them as individuals, commensurate with definitions of ‘person-centred care’. We interpret their sentiment here as ‘good care’.’’ |
| 22 | Social and functional value seemed to also played a large role in the use of the app by HCWs, but this is not reflected in the conceptual framework. Their limited and tentative use in the model is only described as 'PCC logic' in Figure 2, but the paper talks about unmet expectations, misaligned functionality, the burden of engaging with the app, duplication of work, etc. Do the authors feel that the conceptual framework sufficiently describes the HCW’s use case? | We did not frame determinants of HCW as social or functional use as our primary aim was the understand use among patients.  Instead, we have now incorporated factors influencing healthcare workers' engagement with the app. These factors are not entirely new but have been drawn from existing key themes that underpin the first half of the Results section, preceding the “App Uptake and Use Among Patients” subsection.  Please refer to the updated Figure 2 on page 24. |
| 23 | How does time work in the conceptual model? The text describes a gradual decline in app usage over time, but did participants shift categories throughout care? For example, for those classified as being in the performative group, did they start in the functional or enhanced user groups and then convert to performative use by the end of treatment? As their participation changed over time, at what point were they categorized as falling into one of the quadrants? This should be addressed in the methods and results. | Thank you for highlighting the importance of the temporal aspect and possibility of category shifting among patient users, which we initially considered but did not incorporate well enough into the original manuscript.  We have reviewed our documentation of and analysis of patterns of patients’ app use and how our analysis evolved. While we interviewed participants retrospectively about their experiences, we were not able to identify any patient users within our sample who exhibited clear patterns of migration or transition between categories. This could be due to the study’s design (timing, duration, and one-off encounter) which limited our ability to observe such changes over time.  However, this does not imply that app users, especially patients, remain static within a particular category perpetually. Instead, we suggest that with ongoing technology modifications and redesigns, mHealth interventions could facilitate positive user transitions over time—particularly helping users move out of less desirable quadrants, such as performative use or non-use.  To address the insight you suggest, we have added information to the discussion on pages 34-35, lines 633-636: ‘’While we do not diminish the contributions that interventions which convey social value may have, to achieve their intended effect mHealth interventions should be transitioning users toward functional or enhanced use, while shifting them away from performative or unproductive use patterns.’’  To acknowledge that the study design may limit us to unpack such insight, we rewrite the text in the Strength and Limitation section on page 37, lines 681-683: ‘’This study only investigated the early stage of implementation. A mixed-method, and longitudinal design should be considered to extend the interpretation of the findings. |
| 24 | Page 25- The functional use group only included 2 people. Do the authors feel that this is a large enough sample to include a profile of the group? | Your concern is valid and deserves a clearer explanation. We have detailly addressed this point in Comment 17. |
| 25 | I would suggest renaming category 4 in the framework since the adjective ‘unproductive’ could potentially also apply to the performative use category. It sounds more like the digital divide created non-use or family-enabled use. | We agree with your suggestion to change this descriptor to ‘Non-/Reliant Use’ because it (1) helps to delineate different kinds of non-use, and (2) emphasizes further that performative use is the more worrisome group.  We have changed in sub-heading for the fourth category to ‘’Non-use’’ on page 28, line 508. The corresponding descriptor in Figure 2 on page 24 has also changed accordingly. |
| 26 | The discussion does not engage with the literature on Human Centered Design, which addresses many of the iterative design practices for technology/mHealth that are promoted in the paper. | Thank you for your insightful comment. We have added text to further engage with perspectives from Human-Centered Design (HCD). Our rationale is that while this paper aligns with HCD principles, it raises concerns about whether HCD is a meaningful mechanism to integrate mHealth technologies into existing infrastructure and health systems.  Although HCD focuses on individual needs, its impact often remains at the individual level without ensuring these needs are recognized and addressed by the broader local relational needs and practices as well as health systems. This raises the risk of HCD operating in isolation rather than being embedded within the context and systemic structures.  We have now included in the discussion an additional paragraph to make the link between the findings with the literature on Human Centered Design on page 35, lines 638-644: ‘’The app's design employs a human-centered approach as a means to develop mHealth technologies that support patient-centered MDR-TB care (54). There is a risk that this design approach may inadvertently be applied with too narrow a focus on individual user needs and adopt a cross-sectional assessment. This may compromise focus on the complexity of evolving doctor-patient dynamics and the broader healthcare system over time, both of which are important considerations for enhancing patient-centered care (28).’’ |
| 27 | The conclusions should be updated to align with my earlier comments regarding the aim of the paper. | Agree. This is reasonable given the heavy edits that have been made throughout the manuscript.  We have now rewritten the conclusion to reflect the changes made to the aim of the paper on pages 37-38, lines 686-694: ‘’ This qualitative study of mHealth in MDR-TB care in Vietnam reveals the significance of social influence on engagement with digital health technologies. The social value ascribed to the intervention technologies, which was not sustained in light of experienced limited functionality, may provide some explanatory insight into the challenges facing many mHealth interventions. We advocate for an iterative approach, utilizing qualitative inquiry, to development of patient-centered mHealth interventions adaptive to technology-moderated therapeutic dynamics, the local needs and priorities of end-users, as well as the complex organization of care provision in practice.’’ |
| **Reviewer 2** | | |
| 28 | I thank the authors for documenting the use of a digital tool to improve pharmaco-vigilance in an MDR-TB Program in Vietnam. Please receive, below, a few comments for consideration: | Thank you. |
| 29 | (a) the manuscript title is not concise. Please cut out some words and include descriptions that aptly reflect the content of the manuscript such as the study design. The phrase, "Making it fit...., examining the challenges on ... potential..." needs revision | The title has been revised to be more concise and better aligned with the concerns and content of the paper.  We have updated the title to reflect the adjustments made to the paper on page 1 lines 1-4: ‘’Examining the challenges in sustaining user engagement with a mobile app to enhance multidrug-resistant tuberculosis (MDR-TB) care in Vietnam and its implications for implementing person-centred mHealth interventions’’ |
| 30 | (b) In the abstract and introduction sections, the justification for optimal adverse event monitoring in DR-TB programs is not adequately presented. In the introduction, I suggest that the authors provide a rationale for timely adverse event monitoring in the DR-TB program. Please also share statistics on the incidence of the adverse events in Vietnam, and how these have been managed. Provide insights on the WHO guidance and national policies on pharmaco-vigilance in the DR-TB program. This will make the case for the use of digital technologies in these programs. What is the mobile phone penetration rate in Vietnam - is this the same across all the sampled provinces? | We respectfully refute the reviewer on this point. This does not fall under the scope of this study, but much more the larger trial/research under which this study is nested. As we (the qualitative research team) are not part of the main trial team that conceptualized the intervention and our study does not assess its effectiveness, we are not the appropriate researchers to provide the rationale for its design or implementation.  Instead, we have directed readers to the published trial protocol for further details (pages 8-9, lines 154-158 / citation 34). In the discussion, we have emphasized the importance of qualitative research alongside quantitative approaches in evaluating mHealth interventions. |
| 31 | (c) The authors report that they observed that there was a reduction in the use of the app, but there are no numbers presented to support this assertion. | Thank you for this suggestion. We have included statistical output from the trial dashboard which captures cumulative monthly app use by patients enrolled in the intervention arm of the trial.  Page 8, lines 140-143: ‘’This qualitative study sought to explain why although there was initially high uptake, there was an unexpected decline in app engagement (use), with the proportion of patient users checking in at least 5 days/week dropping from 58.6% in the first month (October 2020) to 8.3% by the eleventh month (forthcoming publication).’’ |
| 32 | (d) Did the authors use any theoretical model to guide their qualitative inquiry? | No, we used an inductive analytical approach and developed a theory (the conceptual framework) from the data.  We have clarified our analytical process under the subheading 'Data analysis' on page 15, lines 275-278: ‘’This iterative process of interpretation and conceptualization produced a conceptual framework inductively (40) to explain the observed reduction of app use among patients, which we present within an overall explanatory narrative (41).’’ |
| 33 | (e) In the results section, the authors discuss their findings - this should fit in the discussion section. The results section should answer the question, "What did you find?" | We understand the reviewer’s concern. The interpretative nature of the results may stem from the conceptual framework we developed and, in part, from an unclear description of the analytical process (as noted in Comment 33). Our findings are presented within this framework, which serves as both a lens for analysis and an integral part of our results.  While our Results section may not follow the conventional structure of some other qualitative studies, it is not unusual in some qualitative papers to explain the findings as part of our interpretation within the results section. We consider that adopting this less common but accepted approach is necessary to effectively capture and convey the complexity and nuance of our findings.  Please refer to additional information about our analytical process as per in previous Comment 32 (pages 15, lines 275-278). |
| **Reviewer 3** | | |
| 34 | Intro  - The introduction is quite lengthy and could be more focused. It would benefit from streamlining the background information, emphasizing the specific gap in the literature, and presenting the study's objectives more clearly. Reducing repetition and prioritizing relevant studies will help make the introduction more concise and directly aligned with the research question. | We agree. The introduction has now been streamlined, with a clearer articulation of the gap in the literature and a more precise statement of the paper’s objectives.  Please refer to the edits made in response to Comments 15 and 16. |
| 35 | - In the second paragraph, instead of referring to all drugs used for TB or MDR as antibiotics, it would be more accurate to use the term 'drug regimen' | Thank you for pointing out the term use that may cause confusion. This has been changed as suggested.  The corrected text is located on page 5, line 88. |
| 36 | Result  - describe the sample subsection in the methods section, focusing on participant characteristics in the results section. Move details unrelated to participant characteristics to the methods section. | We also agree with this comment. Details unrelated to participant characteristics have been moved to the methods section.  The following text describing how participants were recruited and interviewed which was previously in the result section has now moved to under Recruitment and Data Collection under the Method section on page 13, lines 237-243: ‘’We selected and approached 28 patients. Three declined due to scheduling conflicts. The 12 HCWs participating in the trial in the three provinces selected as qualitative sites agreed to participate. Individual in-depth interviews were conducted by DT, either via phone (n=18), Zalo (n=12) or in-person (n=4). Once local COVID-19 restrictions were lifted, further interviews were conducted in-person (n=3) to ensure the data collection method was not inadvertently excluding the experiences of those who were not comfortable engaging with the app and/or the study via phone (36).’’ |
| 37 | -Why were significantly more male patients interviewed compared to females, nearly double in number? What was the reason for this discrepancy? | We appreciate the reviewer’s comment and appreciate that it has alerted us that insufficient contextual information was provided for readers. This skewed sex ratio in the sample reflects the global and Vietnam-specific risk factors for MDR-TB, as outlined in the Global TB Report 2024.  We have now included an extra sentence with relevant citations to explain the sex distribution within the sample on page 15, lines 283-285: ‘’The higher number of male patients recruited for the study reflects the disproportionate impact of TB on the male population (6).’’ |
| 38 | - The organization of the results section appears somewhat scattered and does not clearly reflect the themes and sub-themes. I recommend reorganizing the results for better clarity. You could include a table or graphic to summarize the themes and sub-themes related to the challenges faced by both healthcare workers and patients. This would provide a clear overview of the challenges at a glance and make it easier to follow the rest of the results | Our Results section is not structured strictly by themes and sub-themes, as our interpretation of the data extends beyond discrete thematic categories. Instead, we present a conceptual framework within an explanatory narrative that directly addresses the research question while preserving the rich context of the findings.  To improve clarity, we have incorporated a graphic roadmap to visually outline the logic of the Results section, with themes and sub-themes clearly nested within it—thank you for this valuable suggestion.  In developing this roadmap, we may also take the opportunity to refine the organization of the Results section by adding and retitling subheadings.  Please refer to the added Figure 1 on page 17 for the graphic roadmap outlining the logic of the Results section.  We have also added a new sub-heading 'Misalignment with local MDR-TB system solutions and challenges’ on page 21, line 386.  The previous sub-heading ‘How good care facilitated intervention uptake’ has now changed to ‘Initial enthusiasm: how good care facilitated intervention uptake’ on page 23 line 419. |
| 39 | Discussion  -The discussion is rich in content but could benefit from a more streamlined structure to avoid overwhelming the reader | Thank you for the suggestion. To maintain a clear focus, we have streamlined the discussion, ensuring it remains aligned with the paper’s objectives. Additionally, we have introduced subheadings to improve the structure and enhance readability, preventing readers from feeling overwhelmed.  The discussion is now organized as follows:   (1) Summary of the findings (no sub-heading) (pages 31-32, lines 561-578) (2) Lessons for mHealth interventions for MDR-TB care (pages 32-33, lines 579-596) (3) Contribution to conceptualizing the use of mHealth technologies (pages 33-35, lines 597-636) (4) Integrated participatory approach in patient-centred mHealth intervention (page 35, lines 637-652) |
| 40 | - The narrative drifts into broader reflections on mHealth interventions in general | Thank you, in light of this comment we have now contracted our discussion on how the findings relate to wider mHealth intervention into one sub-section under the subheading 'Contribution to mHealth technologies and intervention’.  Please refer to the text under the sub-heading ‘Contribution to conceptualizing the use of mHealth technologies’ on pages 33-34, lines 598-636. |
| 41 | - Certain sentences are overly long and complex, making the content harder to follow | Thank you for this valuable feedback. We have reviewed the manuscript and revised sentences that were overly long or complex to improve clarity and readability. By breaking down lengthy sentences and simplifying the language where appropriate, we hope to ensure that the content is more accessible to readers. We appreciate the reviewer’s suggestion and believe these refinements enhance the overall clarity of the paper.  As the revisions are spread throughout the manuscript, detailing each change may not be feasible within this document. Please refer to the tracked version to review the refinements made to the language. |
| 42 | - Providing suggestions for future studies on how to delve deeper into the social and functional dynamics of mHealth interventions, especially within complex healthcare settings, would be valuable. | Thank you. In revising the manuscript, we have incorporated suggestions for future research, taking into account the methodological limitations of this study.  Additional text has been provided under the Strength and Limitation section on page 37, lines 677-683: ‘’ The study's limitations highlight areas for future research. Including perspectives from those involved in the app's design and development could expand findings to better capture mHealth development dynamics. The financial impact on app use is under-explored. Future research should examine the role of financial incentives on app engagement. This study only investigated the early stage of implementation. A mixed-method, and longitudinal design should be considered to extend the interpretation of the findings.’’ |
| 43 | - lack of actionable insights for policymakers and implementers | We appreciate the reviewer’s recognition of the need for actionable insights presented in the Discussion. In the second half of the revised Discussion, we have intentionally structured our recommendations to provide clearer and more practical guidance for future mHealth interventions. Given the nature and scope of this study, our recommendations primarily focus on evaluating mHealth interventions, offering insights for researchers, practitioners, and policymakers in this field. For broader, upstream policy actions, our findings should be considered within the broader body of mHealth literature rather than in isolation.  To further enhance clarity and accessibility, as per Comment 29, we have refined the structure of the Discussion section, ensuring that the recommendations are well-organized and directly aligned with the study’s key insights.  Kindly refer to the edit in Comment 39 as these edits also implicitly provide recommendations for a range of stakeholders working in Health. |
